# Superior Energy Density Achieved in Unfilled Tungsten Bronze Ferroelectrics via Multiscale Regulation Strategy

**DOI:** 10.1002/advs.202300227

**Published:** 2023-04-21

**Authors:** Haonan Peng, Zhen Liu, Zhengqian Fu, Kai Dai, Zhongqian Lv, Shaobo Guo, Zhigao Hu, Fangfang Xu, Genshui Wang

**Affiliations:** ^1^ Key Laboratory of Inorganic Functional Materials and Devices Shanghai Institute of Ceramics Chinese Academy of Sciences Shanghai 200050 P. R. China; ^2^ Center of Materials Science and Optoelectronics Engineering University of Chinese Academy of Sciences Beijing 100049 P. R. China; ^3^ State Key Laboratory of High Performance Ceramics and Superfine Microstructures Shanghai Institute of Ceramics Chinese Academy of Sciences Shanghai 200050 P. R. China; ^4^ Technical Center for Multifunctional Magneto‐Optical Spectroscopy (Shanghai) Engineering Research Center of Nanophotonics & Advanced Instrument (Ministry of Education) Department of Materials School of Physics and Electronic Science East China Normal University Shanghai 200241 P. R. China; ^5^ School of Chemistry and Materials Science Hangzhou Institute for Advanced Study University of Chinese Academy of Sciences Hangzhou 310024 P. R. China

**Keywords:** charge–discharging, energy storage density, multiscale regulation strategy, polar nanoregion, tungsten bronze ferroelectrics

## Abstract

The most promising candidates for energy storage capacitor application are relaxor ferroelectrics, among which, the perovskite structure ferroelectric ceramics have witnessed great development progress. However, less attention has been paid on tetragonal tungsten bronze structure (TTBS) ceramics because of their lower breakdown strength and polarization. Herein, a multiscale regulation strategy is proposed to tune the energy storage performances (ESP) of TTBS ceramics from grain, domain, and macroscopic scale. The enhanced relaxor behavior with dynamic polar nanodomains guarantees low remanent polarization, while the refined grains and enlarged bandgap ensure increased breakdown strength. Hence, excellent ESP is realized in unfilled TTBS Sr_0.425_La_0.1_□_0.05_Ba_0.425_Nb_1.4_Ta_0.6_O_6_ (SLBNT) ceramics with an ultrahigh recoverable energy density of 5.895 J cm^−3^ and a high efficiency of 85.37%. This achievement notably surpasses previous studies in TTBS ceramics and is comparable to that of perovskite components. Meanwhile, the energy density exhibits a wide temperature, frequency, and cycling fatigue stability. In addition, high power density (257.89 MW cm^−3^), especially the ultrafast discharge time (*t*_0.9_ = 16.4 ns) are achieved. The multiscale regulation strategy unlocks the energy storage potential of TTBS ceramics and thus highlights TTBS ceramics as promising candidates for energy storage, like perovskite structured ceramics.

## Graphical abstract

An ultrahigh recoverable energy density of 5.895 J cm^−3^ is realized for the first time in tetragonal tungsten bronze structure (TTBS) ceramics through multiscale regulation from grain scale, domain scale, and macroscopic scale. This work uncovers the energy storage potential of TTBS ceramics and underlines them as promising candidates for energy storage, like the perovskite structure ceramics.

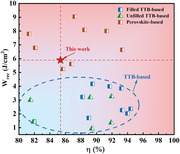

## 1 Introduction

Dielectric capacitors have received increasing attention as the heart of pulse power systems due to their fast charge–discharge capability and high power density. Nonetheless, dielectric capacitors take up over 25% of the volume and weight of pulse power systems,^[^ ^1^ ^]^ which contradicts the development trend toward miniaturization and integration for modern electrical and electronic devices. Consequently, achieving high energy storage properties in dielectric materials is identified as the major obstacle to the development of high‐end pulse power capacitors. And the energy storage performance (ESP) can be determined by integrating the polarization–electric field (*P*–*E*) hysteresis loops: $W_{\mathrm{rec}\;}=\int_{P_{r}}^{P_{m}}EdP$, $W_{\mathrm{total}\;}=\int_{0}^{P_{m}}EdP$,$\;\eta =\frac{W_{\mathrm{rec}\;}}{W_{\mathrm{total}\;}}\times \;100\%$, where *W*_rec_, *W*_total_, *η*, *P*_m_, *P*_r_, and *E* represent recoverable energy storage density, total energy storage density, energy storage efficiency, maximum polarization, remanent polarization, as well as the applied electric field, correspondingly.^[^ ^2^, ^3^ ^]^ Hence, a high *P*_m_, low *P*_r_, and a high dielectric breakdown strength (BDS) are critical factors for achieving high *W*_rec_ and *η*. In the meantime, rapid response to an applied electric field, delayed saturation polarization, uniform and dense microstructure, refined grains, and a large bandgap for carrier transfer are required in high‐performance dielectric ceramics.^[^ ^4^ ^]^

Dielectric ceramics can be divided into four typical types: linear dielectric materials (LD), normal ferroelectrics (FEs), antiferroelectric (AFEs), and relaxor ferroelectrics (RFEs).^[^ ^5^ ^]^ RFEs with high dynamic and weak coupling polar nanoregions (PNRs) seem to be the optimal material for energy storage. And the weakly coupled PNRs are electric field‐sensitive and respond quickly to the variation of external electric field, thus gaining a high *P*_m_ and a negligible *P*_r_,^[^ ^6^, ^7^ ^]^ which will result in a large Δ*P* (*P*_m_ – *P*_r_) and ensure the achievements of great *W*_rec_ and*η* in RFEs. Over the past decade, multiple strategies have been investigated to strengthen the ESP of perovskite structure RFEs, including nanodomain design/engineering,^[^ ^5^, ^8^, ^9^ ^]^ superparaelectric design,^[^ ^10^, ^11^, ^12^ ^]^ and defect engineering^[^ ^13^ ^]^ et al. Hence, high *W*_rec_ (typically higher than 4 J cm^−3^) and *η* (no less than 80%) are achieved in all types of perovskite components, such as BaTiO_3_‐based (0.6BaTiO_3_‐0.4Bi(Mg_0.5_Ti_0.5_)O_3_, 4.49 J cm^−3^ and 93%),^[^ ^14^ ^]^ NaNbO_3_‐based (0.68NaNbO_3_‐0.32(Bi_0.5_Li_0.5_)TiO_3_, 8.73 J cm^−3^ and 80.1%),^[^ ^15^ ^]^ Bi_0.5_Na_0.5_TiO_3_‐based (0.65(0.84Bi_0.5_Na_0.5_TiO_3_‐0.16K_0.5_Bi_0.5_TiO_3_)‐0.35(Bi_0.2_Sr_0.7_TiO_3_), 4.06 J cm^−3^ and 87.3%),^[^ ^16^ ^]^ BiFeO_3_‐based (0.57BiFeO_3_‐0.33BaTiO_3_‐0.1NaNbO_3_, 8.12 J cm^−3^ and 90%),^[^ ^5^ ^]^ and K_0.5_Na_0.5_NbO_3_‐based (0.85K_0.5_Na_0.5_NbO_3_‐0.15Bi(Ni_0.5_Zr_0.5_)O_3_, 8.09 J cm^−3^ and 88.46%) ceramics.^[^ ^17^ ^]^

Tetragonal tungsten bronze structure (TTBS) ferroelectric ceramics are well‐known as the second largest family of ferroelectrics after the perovskite ferroelectrics. However, TTBS ceramics have received much less attention for energy storage study due to their low saturated polarization and poor BDS related to abnormal grain growth.^[^ ^18^ ^]^ The connections between the BO_6_ octahedron's common vertices form the framework of TTBS ceramics and three types of interstices: square A1, pentagonal A2, and triangular C sites.^[^ ^19^ ^]^ And subsequently, TTBS ceramics are categorized into three groups on the basis of the ion occupancy situation: fully filled (A and C sites are all occupied), filled (all A sites are occupied, C sites are empty), and unfilled (A sites are partially occupied, C sites are empty).^[^ ^20^, ^21^ ^]^ Some investigations into TTBS ceramics for energy storage have been conducted, but the *W*_rec_ is frequently unsatisfactory (less than 4 J cm^−3^). For instance, Sr_0.5_Na_0.5_Nb_2_O_5_‐based ((Sr_2_NaNb_3.5_Ta_1.5_O_6_ (*W*_rec_ = 3.99 J cm^−3^ and *η* = 91.7%)),^[^ ^22^ ^]^ Sr_0.5_K_0.5_Nb_2_O_5_‐based (Sr_2_KNb_4.2_Ta_0.8_O_15_ (*W*_rec_ = 3.84 J cm^−3^ and *η* = 93.2%)),^[^ ^23^ ^]^ and Sr_0.5_Ba_5_Nb_2_O_6_‐based (0.7Sr_0.75_Ba_0.25_Nb_2_O_6_‐0.3PbZr_0.52_Ti_0.48_O_3_ (*W*_rec_ = 3.0 J cm^−3^ and *η* = 81.5%) ^[^ ^24^ ^]^). Prior research has established that the ion doping modulated regularity of ferroelectric and relaxor behavior in TTBS ceramics, which is primarily a result of cation occupancy and structural distortion.^[^ ^25^, ^26^, ^27^, ^28^, ^29^, ^30^, ^31^ ^]^ In accordance with the crystal‐chemical framework, a small A‐site average ion radius causes a reduction in O—B—O bond length and benefits the improvement of relaxor properties.^[^ ^32^ ^]^ Meanwhile, the weakened B—O bonding, local structural fluctuation or distortion, and order–disorder distribution all support the modification of the B‐site cation to improve relaxor behavior.^[^ ^29^, ^33^, ^34^ ^]^ Additionally, the introduction of A‐site vacancies can induce extra changes in the local coordination environment, structure relaxation, and charge/structure disorder.^[^ ^26^, ^35^, ^36^, ^37^ ^]^ Consequently, various methods can be adopted to adjust the relaxor behaviors of TTBS ceramics, and their flexible structures and abundant properties tunability provide a suitable realm for the effective design of high energy storage performance.^[^ ^38^ ^]^ Hence, the TTBS ceramic family merits more attention and investigation as an energy storage material. The innovative design of high ESP TTBS ceramics would assist in further comprehending the regulation mechanism between composition, structure, and properties, and may open a new window for future development of high performance energy storage materials.

**Figure 1:**
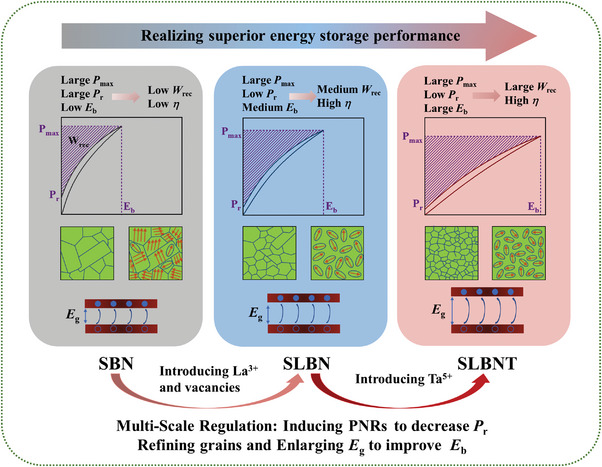
Schematic diagram of multiscale regulation strategy to improve the energy storage performance of Sr_0.5_Ba_0.5_Nb_2_O_5_ unfilled tungsten bronze structure ceramics.

In this work, we proposed a multiscale regulation strategy to improve the energy storage performance of Sr_0.5_Ba_0.5_Nb_2_O_5_ unfilled tungsten bronze structure ceramics (**Figure** **1**). Via La^3+^ and Ta^5+^ doping, the designed Sr_0.425_La_0.1_□_0.05_Ba_0.425_Nb_1.4_Ta_0.6_O_6_ (SLBNT) ceramics achieved enhanced relaxor behavior by destroying the ferroelectric long‐range order and inducing polar nanoregions at domain scale. Extra vacancies were also introduced to satisfy electrical neutrality and further facilitate structural relaxation and disorder. In addition, the substitution of large bandgap oxides Ta_2_O_5_ (≈4 eV) and La_2_O_3_ (≈4.3 eV) can enlarge the bandgap. Meanwhile, the strong refractory nature of Ta^5+^ can significantly inhibit grain growth and reduce the average grain size. The refined grains and enlarged bandgap from the grain scale and macroscopic scale, guarantee together an increased *E*_b_. Ultimately, the SLBNT ceramics demonstrated excellent energy storage performance (*W*_rec_ = 5.895 J cm^−3^ and *η* = 85.37%), which was superior to all previous reports on TTBS ceramics. And the high power density (257.89 MW cm^−3^) and ultrafast discharge time (*t*_0.9_ = 16.4 ns) further incorporated the potential of SLBNT ceramics for application. This work developed a novel lead‐free preferential material for pulse power applications and highlighted the research prospects of TTBS ceramics for energy storage.

## 2 Results and Discussion

**Figure 2:**
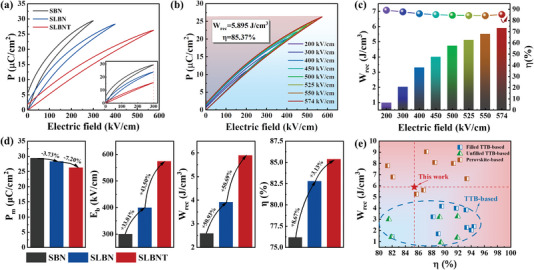
a) The *P–E* loops of SBN, SLBN, and SLBNT ceramics under respective *E*_b_ (the inset shows the *P–E* loops under 300 kV cm^−1^). b) The *P–E* loops of SLBNT ceramics under different electric fields. c) *W*_rec_ and *η* values of SLBNT ceramics under different electric fields. d) A comparison of *E*_b_, *P*_m_, *W*_rec_, and *η* values between SBN, SLBN, and SLBNT ceramics. e) The comparison of *W*_rec_ and *η* values of SLBNT and other recently reported lead‐free bulk ceramics.^[^ ^17^, ^18^, ^22^, ^23^, ^24^, ^27^, ^28^, ^29^, ^38^, ^39^, ^40^, ^41^, ^42^, ^43^, ^44^, ^45^, ^46^, ^47^, ^48^, ^49^, ^50^, ^51^, ^52^, ^53^ ^]^

**Figure** **2**a illustrates the *P*–*E* loops of Sr_0.5_Ba_0.5_Nb_2_O_6_, Sr_0.425_La_0.1_□_0.05_Ba_0.425_Nb_2_O_6_, and Sr_0.425_La_0.1_□_0.05_Ba_0.425_Nb_1.4_Ta_0.6_O_6_ ceramics (abbreviated as SBN, SLBN, and SLBNT, correspondingly) recorded under their corresponding BDS and a frequency of 10 Hz. With the variation in composition, the *P*_m_ decreases monotonously, as also can be seen in Figure 2d and the inset of Figure 2a, which demonstrates the composition dependent *P*–*E* loops at fixed *E* of 300 kV cm^−1^. The BDS of the samples, however, increases evidently, from ≈300 kV cm^−1^ for SBN to ≈400 kV cm^−1^ for SLBN, and ultimately reaches ≈574 kV cm^−1^ for SLBNT (Figure 2d) with the introduction of La^3+^ and Ta^5+^. Results from the codoping strategy, a steadily enhanced relaxor character with slimmer *P–E* loops and lower *P*_r_ has also been realized, as revealed from the *E* dependent *P–E* loops in Figure 2b. The ESPs of three compositions are shown in Figure 2d. SBN ceramics present a poor ESP (*W*_rec_ = 2.592 J cm^−3^ and *η* = 76.18%) due to the comparatively large *P*_r_ and low *E*_b_. For SLBN, despite the fact that an enhancement of 33.01% was realized in *E*_b_, the *W*_rec_ is nevertheless lower than 4 J cm^−3^. Finally, the designed SLBNT ceramics achieve an ultrahigh *E*_b_ of ≈574 kV cm^−1^ with a further increase of 43.5%. As a result, *W*_rec_ increases to 5.895 J cm^−3^ with an enhancement of 50.69%, and *η* is also improved to 85.37%. The achievement of optimized composition of Sr_0.425_La_0.1_□_0.05_Ba_0.425_Nb_1.4_Ta_0.6_O_6_ can also be supported by a systematical investigation on the structure and properties of Sr_0.425_La_0.1_□_0.05_Ba_0.425_Nb_2−_ *_x_* Ta*_x_* O_6_ ceramics with different Ta^5+^ concentrations, which is summarized in Figures S1–S4 (Supporting Information). A comparison of the ESP of our SLBNT ceramics and previously reported lead‐free bulk ceramics are illustrated in Figure 2e. It is evident that the excellent overall performance of SLBNT ceramics leads the field in tetragonal tungsten bronze structure ferroelectric ceramics and can even be comparable with some state‐of‐the‐art perovskite structure ceramics.^[^ ^17^, ^18^, ^22^, ^23^, ^24^, ^27^, ^28^, ^29^, ^38^, ^39^, ^40^, ^41^, ^42^, ^43^, ^44^, ^45^, ^46^, ^47^, ^48^, ^49^, ^50^, ^51^, ^52^, ^53^ ^]^

**Figure 3:**
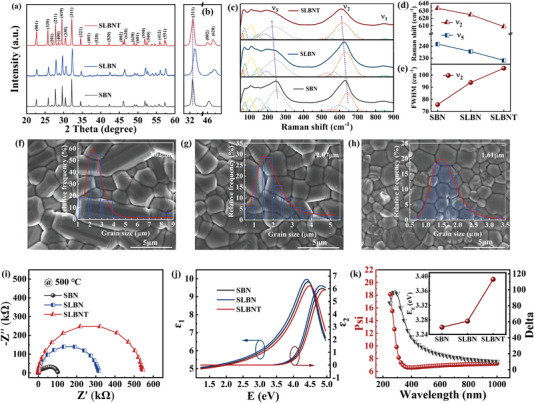
a) XRD patterns of the SBN, SLBN, and SLBNT ceramics at room temperature. b) Enlarged views of (311), (002), and (620) peak. c) Raman spectra of SBN, SLBN, and SLBNT ceramics at room temperature. d) Raman shift of *ν*_2_ and *ν*_5_ modes and e) FWHM of *ν*_2_ mode as a function of composition. SEM images and grain size distributions of f) SBN, g) SLBN, and h) SLBNT ceramics. i) Impedance spectra of SBN, SLBN, and SLBNT ceramics at 500 °C. j) The optical dielectric constant as a function of phonon energy. k) the experimental ellipsometric (dots) and the best‐fitting (solid lines) spectra of SLBNT ceramics (the inset shows the *E*_g_ of all components).

**Figure** **3**a shows the X‐ray diffractometer (XRD) patterns of SBN, SLBN, and SLBNT ceramics. All the patterns reveal pure tungsten bronze structure, indicating that the La^3+^ and Ta^5+^ successfully entered the SBN host lattice. The magnified patterns around 32° and 46° are presented in Figure 3b. Due to the smaller ionic radius and higher electronegativity, La^3+^(1.36 Å (12 coordination), 1.1) occupies the A1 site like Sr^2+^(1.44 Å (12 coordination), 0.95). As a direct consequence, the unit cell shrinks, and the lattice parameters decline as a result of decreasing ion radius, let alone the formation of extra A‐site vacancies due to aliovalent ion substitution. The impact of extra vacancy is concluded as an important local distortion: a Nb/Ta cube around a vacancy is compressed in *c* axis, which means a larger tilts of octahedra.^[^ ^54^ ^]^ Accordingly, the diffraction peaks slightly shift to higher 2*θ* values from SBN to SLBN. On the contrary, the diffraction peaks of SLBNT show a pronounced shift toward lower 2*θ* value although Ta^5+^(0.64 Å (12 coordination)) and Nb^5+^(0.64 Å (12 coordination)) have same ion radii. This shift in diffraction peaks could be attributed to the difference in electronegativity between Ta^5+^ (1.51) and Nb^5+^ (1.59), which leads to a weaker covalent bond.^[^ ^33^ ^]^ According to the second‐order Jahn–Teller effect associated with the electron orbital hybridization between B‐site cations and oxygen, the ferroelectricity of TTB originates from the displacement of the B‐site ion along the center of the octahedron.^[^ ^25^ ^]^ While the different electron configurations of Ta^5+^ can directly change the displacement of polar cations, resulting in the tilting of the oxygen octahedron and enhanced relaxor behaviors. Moreover, the diffraction peak around 46° steadily split into two peaks (the low‐angle (002) peak and the high‐angle (620) peak). This splitting of diffraction peaks is ascribed to the reduced tetragonality (c/a). The more accurate Rietveld refinement is illustrated in Figure S5 (Supporting Information), and the crystallographic data are summarized in Table S1 (Supporting Information). It shows that all compositions belong to the tetragonal crystal system with a space group of *P4bm*, and the tetragonality (c/a) decreases with the ions doping. Meanwhile, compression of structural deformation along the polar *c* axis (a decreasing lattice parameter c) points to the suppressed ferroelectric nature, which means that the decreased tetragonality would be related to the relaxor behavior. The decreased tetragonality disturbs the displacement of the B‐site ferroelectric active ion along the center of the octahedron, resulting in strong relaxation of SLBNT.^[^ ^25^, ^27^, ^36^ ^]^

The local structures of SBN, SLBN, and SLBNT ceramics were also examined by using Raman spectroscopy. Figure 3c depicts three typical internal vibration modes: *ν*_1_ mode (≈840 cm^−1^) and *ν*_2_ mode (630 cm^−1^) related to the O—B—O bending vibrations, and the B—O (*ν*_5_) stretching vibrations mode at ≈250 cm^−1^. Other vibration modes below 200 cm^−1^ are external vibrations associated with the motions of the A site cation.^[^ ^49^ ^]^ Smaller‐ion‐radius La^3+^ can increase stretching vibrations while lessening the interaction between metal ions and oxygen.^[^ ^55^ ^]^ Moreover, the addition of Ta^5+^ can also weaken the strong interaction because of its different electronic configuration from Nb^5+^.^[^ ^52^ ^]^ Consequently, the *ν*_2_ and *ν*_5_ modes exhibit a redshift attributable to the weakened covalency of the B—O bond and the expansion of the BO_6_ octahedron, as displayed in Figure 3d.^[^ ^51^ ^]^ The increased full width at half maximum (FWHM) of *ν*_2_ mode in Figure 3e also indicates the enhanced disorder in short‐range structure and the increasing distortion degree of the BO_6_ octahedron.^[^ ^49^ ^]^ In a word, these variations and distortions of the BO_6_ polar unit are affected by La^3+^ and Ta^5+^ in different ways, whereas, both contribute to local polarization and enhanced relaxor characteristic.

The microstructures of SBN, SLBNT, and SLBNT ceramics are displayed in Figure 3f–h, and the insets show the grain size distribution. SBN presents an inhomogeneous microstructure with anisometric and columnar‐shaped grain, which is caused by the faster growth rate along the (001) facet.^[^ ^34^ ^]^ The amount of columnar‐shaped grain decreases noticeably in SLBN and then disappears in SLBNT. The average grain size (*G*_a_) decreases from 3.02 µm (SBN) to 2.07 µm (SLBN) and eventually reduces to 1.61 µm (SLBNT). The refined grains can be ascribed to the reduction of sintering temperature from 1370 °C for SBN to 1230 and 1300 °C for SLBN and SLBNT, respectively, which might originate from the lattice activation after La^3+^ doping.^[^ ^5^ ^]^ Moreover, the refractory nature of Ta^5+^ can significantly inhibit grain growth, which finally reduces the average grain size and increases the proportion of equiaxed grains for SLBNT.^[^ ^22^, ^23^ ^]^ In accordance with the relationship between *G*_a_ and *E*_b_, described as *E*_B_∝(*G_a_*)^−*a*^,^[^ ^56^ ^]^ the grain boundaries contain depletion layers having higher barriers for charge delivery,^[^ ^57^ ^]^ thus reducing the overall ceramics’ conductivity. Moreover, as seen in Figure 3i; and Figure S6 (Supporting Information), the resistance increases with the decrease of *G*_a_ from SBN to SLBN, and SLBNT. The impedance spectra with two semicircles represents the impedance response of grains and grain boundaries, respectively. The *G*_a_ of SLBN and SLBNT decrease obviously, resulting in more depletion regions and higher grain boundary resistivity. It means that the contribution of grain boundary to resistivity is more evident. Consequently, the SLBN and SLBNT show only one semicircle in impedance spectra.^[^ ^1^, ^58^ ^]^ Figure 3j presents the dielectric functions (*ε* = *ε*_1_ + *ε*_2_) measured by spectroscopic ellipsometry. The experimental and simulation spectra match well through the Tauc–Lorentz (TL) model as shown in Figure 3k.^[^ ^59^ ^]^ With the substitution of large bandgap oxide Ta_2_O_5_ (≈4 eV) and La_2_O_3_ (≈4.3 eV), the calculated *E*_g_ increased from 3.26 eV of SBN to 3.28 eV of SLBN, and 3.39 eV of SLBNT. The large bandgap makes it more difficult for the electrons to transmit from the valence band to the conduction band, reducing the probability of electron ionization and collision during the breakdown process, which can further boost the BDS.^[^ ^52^, ^60^, ^61^, ^62^ ^]^ To sum up, the refined grains and enlarged band gap demonstrate the effectiveness of our regulation strategy of ESP at the grain scale and macroscopic scale.

**Figure 4:**
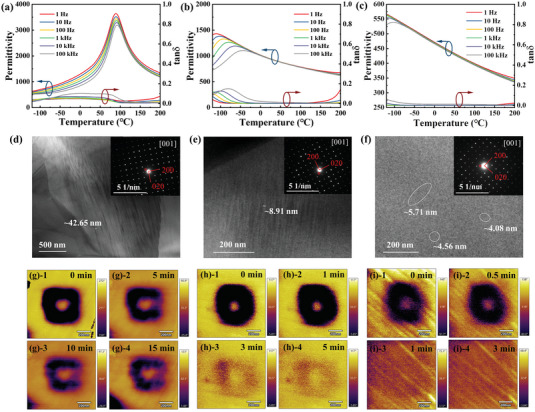
Temperature dependent permittivity and dielectric loss of a) SBN, b) SLBN, and c) SLBNT ceramics under various frequencies. The TEM images of the domain morphology of d) SBN, e) SLBN, and f) SLBNT ceramics (The insets show the SAED patterns along [001] zone axis). The out of plane phase image of g)‐1 to 4 SBN, h)‐1 to 4 SLBN, and i)‐1 to 4 SLBNT ceramics after a voltage of 15 V.

The temperature dependent dielectric permittivity (*ε*_m_) and dielectric loss (tan*δ*) are shown in **Figure** **4**a–c over the range of −120 to 200 °C. In distinction to SBN's sharp and frequency‐insensitive dielectric peak, as frequency increases, the dielectric peaks of SLBN and SLBNT are broad and decrease with increasing temperature. Meanwhile, the decrement of *T*_m_ to below 0 °C indicates the appearance of PNRs at room temperature. Consequently, SLBN and SLBNT ceramics present slimmer *P–E* loops than pure SBN ceramics. The introduction of La^3+^ and Ta^5+^ causes ionic disorder and compositional fluctuation, destroying the long‐range‐ordered ferroelectric microdomains and decreasing the size of domains.^[^ ^21^, ^37^, ^63^ ^]^ Besides, extra structural vacancies at the A‐site further weaken the coupling of PNRs, thereby influencing the relaxor behavior.^[^ ^51^ ^]^ Additionally, the enhanced relaxor behavior is also quantitatively estimated by the calculated diffuseness degree (*γ*) in Figure S7 (Supporting Information) via the Curie–Weiss law ($\frac{1}{\varepsilon_{r}}-\frac{1}{\varepsilon_{m}}=\frac{{\left(T-T_{m}\right)}^{\gamma }}{C}$, where *ε*_m_ is the maximum permittivity and C is a constant).^[^ ^64^ ^]^ The value of *γ* rises dramatically and monotonously from SBN (1.19) to SLBN (1.79) and SLBNT (1.94). And it agrees with the variations of *P–E* loops in Figure 2a, where *P*_r_ decreases with the increase of *γ*. For the purpose of further illustrating the domain structure, the transmission electron microscopy (TEM) analysis was carried out and the results were displayed in Figure 4d–f. The SAED (selected area electron diffraction) patterns along [001] zone axis in the insets of Figure 4d–f show the basic reflections of TTBS ceramics with tetragonal symmetry, and no obvious structural changes are found among SBN, SLBN, and SLBNT ceramics. The domain morphology shows that SBN ceramics possess a large domain size of about 42 nm. The ferroelectric domains are then destroyed in the SLBN ceramics, and domains with small sizes of ≈8.91 nm are formed. Although the contrast is a little less sharp for the SLBNT ceramics, the presence of nanoscale PNRs can be seen in the white circle area. As shown in Figure S8a (Supporting Information), no large size ferroelectric domains can be found in the larger field of view. And at higher magnification (Figure S8c (Supporting Information), PNRs of ≈3.69 nm are visible. Meanwhile, the dynamic response of domain is investigated by the piezoresponse force microscopy (PFM). Figure 4g–i; and Figure S9 (Supporting Information) illustrate the out of plane phase and amplitude images of SBN, SLBN, and SLBNT ceramics after a voltage of 15 V. Long‐range ordered FE domains with strong and clear contrasts still can be observed in SBN after 15 min. For SLBN, the PFM signals fade faster with a relaxation time of 3 min. In contrast, the poled region and piezoresponse of SLBNT fade very fast (1 min) owing to the high dynamic PNRs, which is consistent with typical relaxor behavior.^[^ ^65^ ^]^ In summary, the decreased domain size favors easier polarization reorientation after the external electric field removal and thus benefits for smaller *P*_r_ and slimmer *P*–*E* loops.

**Figure 5:**
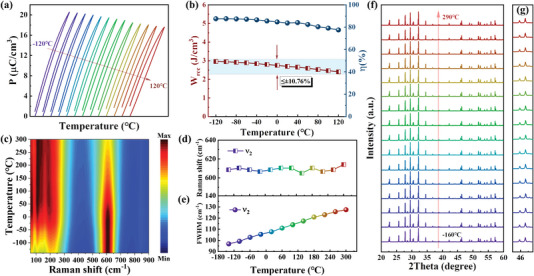
a) The *P–E* loops of SLBNT ceramics at different temperatures. b) *W*_rec_ and *η* as a function of temperature for SLBNT ceramics. c) Temperature‐dependent Raman spectra of SLBNT ceramics. d) The Raman shift and e) FWHM of *ν*_2_ mode as a function of temperature. f) The temperature‐dependent XRD patterns and g) the magnified patterns around 46° of SLBNT ceramics.

The temperature stability of energy storage properties plays a vital role in practical applications. **Figure** **5**a displays the *P*–*E* loops of SLBNT ceramics over a wide temperature range from −120 to 120 °C at 350 kV cm^−1^ and 10 Hz. the *P*–*E* loops of SLBNT ceramics remain slim as the temperature increases. Furthermore, the *P*_m_ and *P*_r_ exhibit a small variation of 2.969 and 1.085 µC cm^−2^, correspondingly. In consequence, the *W*_rec_ shows a slight decline from 2.960 J cm^−3^ at −120 °C to 2.405 J cm^−3^ at 120 °C with a variation of less than ±10.76%, and the *η* decreases from 87.15% to 77.55%, indicating the relatively satisfying temperature stability. Local structure changes under temperature variation in SLBNT ceramics are revealed using temperature‐dependent Raman spectroscopy. As seen in Figure 5c, the number of Raman peaks is unaffected by changes in temperature, indicating that SLBNT ceramics have a stable phase structure.^[^ ^66^ ^]^ As the temperature rises from −140 to 300 °C, the peak intensity of external vibration modes (associated with the motions of the A site cation) below 200 cm^−1^ gradually increases. The enhanced motion of A site ions has a huge impact on the tilting of the BO_6_ octahedron, which is related to the displacement of the B‐site ion along the *c* axis.^[^ ^55^, ^67^ ^]^ As shown in Figure 5d, the *ν*_2_ mode, which originates from the O—B—O bending vibration of the BO_6_ octahedron, exhibits negligible red or blueshifts and only fluctuates slightly around ≈608 cm^−1^. In addition, its peak intensity exhibits a decreasing tendency and its full width at half maximum (FWHM) gradually increases throughout the test temperature range (Figure 5e). These structural features prove suppressed polarity and a higher disorder degree in local structure, ^[^ ^15^, ^29^, ^68^ ^]^ which further confirms the existence of nanodomains in a wide *T* range. Figure 5f illustrates the temperature dependent XRD patterns of SLBNT ceramics in the range of −160–290 °C. Invariant diffraction peaks are evident which supports the stable structure of SLBNT in the whole *T* range. The magnified parts around 46° are also given in Figure 5g. The splitting (002) and (620) peaks display no detectable changes from −160 to 290 °C, suggesting the invariant *P4bm* symmetry upon heating and cooling. The temperature stable structure, as shown by the above Raman and XRD analyses, guarantees the reliability of SLBNT to work in harsh environments under high temperatures or below‐zero temperatures.

**Figure 6:**
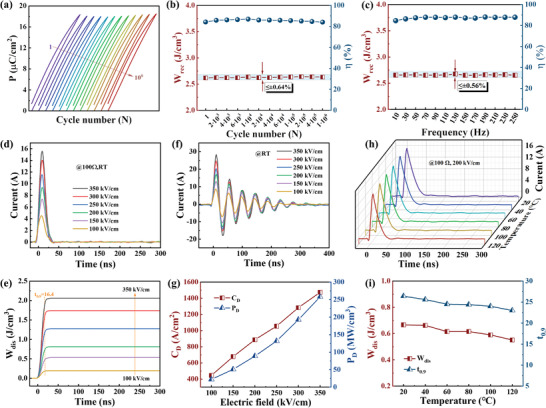
a) The *P–E* loops of SLBNT ceramics at different cycle numbers. The *W*_rec_ and *η* of SLBNT ceramics at different b) cycle numbers and c) frequencies. The room‐temperature d) overdamped discharge and f) underdamped discharge curves of SLBNT ceramics at 100*–*350 kV cm^−1^. e) The *W*_dis_ of SLBNT ceramics as a function of time. g) *C*_D_, and *P*_D_ values of SLBNT ceramics as a function of electric field. h) The overdamped discharge curves, i) *W*_dis_ and *t*_0.9_ of SLBNT ceramics as a function of temperature.

The *P*–*E* loops for SLBNT ceramics are also evaluated at 350 kV cm^−1^ and room temperature under a wide range of cycle numbers and frequencies to further assess the stability of its ESP. **Figure** **6**a,b; and Figure S10(a) (Supporting Information) provide the *P*–*E* loops, *W*_rec_, *η*, and the corresponding *P*_m_, *P*_r_, Δ*P* after 10^6^ cycles. The *P*_m_ and *P*_r_ slightly decrease at first and subsequently increase with the accumulation of cycles. And the *W*_rec_ maintains its current value of about 2.630 J cm^−3^, accompanied by a negligible change (±0.64%). As displayed in Figure S10(b,c) (Supporting Information); and Figure 6c, the SLBNT ceramics preserve slim *P*–*E* loops with low hysteresis loss in the range 10–250 Hz, indicating high frequency stability. The variations of *P*_m_ and *P*_r_ are as small as 0.45 and 0.33 µC cm^−2^, respectively. The *W*_rec_ only slightly decreases from 2.657 J cm^−3^ at 10 Hz to 2.653 J cm^−3^ at 250 Hz, exhibiting a variation of less than ±0.56%.

The charge–discharge tests can be used to determine the discharge properties of SLBNT ceramics for practical applications. The overdamped discharge current curves at room temperature are displayed in Figure 6d. The discharge energy density (*W*_dis_) is calculated from the equation: $W_{\mathrm{dis}}=\frac{R\int i^{2}\left(t\right)dt}{V}$.^[^ ^45^ ^]^ The integral curve is plotted in Figure 6e with a *W*_dis_ of 2.061 J cm^−3^ and an ultrafast *t*_0.9_ of 16.4 ns. Figure 6f presents the underdamped discharge waveforms of SLBNT ceramics at room temperature. The value of current peaks increases linearly as electric fields increase linearly. The current density (*C*_D_) and power density (*P*_D_) were calculated according to following equations: *C*_D_ = *I*_max_/*S*, *P*_D_ = *EI*_max_/2*S*.^[^ ^69^ ^]^ As shown in Figure 6g, the *C*_D_ exhibits the same increasing tendency as *I*_max_, reaching the maximum values of 1473.68 A cm^−2^ and 28.32 A at 350 kV cm^−1^. And the *P*_D_ increases from 22.26 to 257.89 MW cm^−3^. Figure 6h,i examines the temperature stability of the discharge properties at 200 kV cm^−1^ from 20 to 120 °C. The discharge curve remains unchanged, with a slight reduction in peak current. Thus, the calculated *W*_dis_ only exhibits a slight decrease from 0.667 to 0.551 J cm^−3^. Besides, *t*_0.9_ is measured to be shorter than 26.4 ns in the whole test temperature range, and the slight increase of *t*_0.9_ is attributed that the reaction speed of PNRs to electric fields is improved by the enhanced random electric field. The excellent pulse discharging properties under different electric fields and temperatures, as well as the outstanding stability, further emphasize the promise of SLBNT for practical pulse power capacitor applications.

## 3 Conclusion

In conclusion, we proposed a multiscale regulation strategy for attaining excellent energy storage performance in unfilled TTBS relaxor ferroelectrics. Benefiting from the induced polar nanoregions, refined grains, as well as enlarged bandgap via La^3+^ and Ta^5+^ doping, the Sr_0.425_La_0.1_□_0.05_Ba_0.425_Nb_1.4_Ta_0.6_O_6_ ceramics simultaneously realize a record‐high *W*_rec_ among TTBS ceramics of 5.895 J cm^−3^ and a high *η* of 85.37% accompanied with an ultrahigh *E*_b_ of ≈574 kV cm^−1^. Meanwhile, excellent frequency stability (10–250 Hz), cycling fatigue stability (up to 10^6^ times), wide temperature stability (−120–120 °C), high power density (*P*_D_ = 257.89 MW cm^−3^), and ultrafast discharge rate (*t*_0.9_ = 16.4 ns) are likewise demonstrated in the designed ceramics. The overall energy storage performance can also be comparable with the extensively‐studied perovskite structured ferroelectric materials. This work not only provides a promising candidate for high‐performance pulse power capacitors but also underlines the energy storage potential of tetragonal tungsten bronze structure relaxor ferroelectric materials.

## 4 Experimental Section

### Sample Preparation

The Sr_0.5_Ba_0.5_Nb_2_O_6_, Sr_0.425_La_0.1_□_0.05_Ba_0.425_Nb_2_O_6_, and Sr_0.425_La_0.1_□_0.05_Ba_0.425_Nb_1.4_Ta_0.6_O_6_ ceramics (abbreviated as SBN, SLBN and SLBNT, respectively) were fabricated by a traditional solid‐state method. The raw materials SrCO_3_ (99.99%), BaCO_3_ (99.95%), Nb_2_O_5_ (99.99%), La_2_O_3_ (99.95%), Ta_2_O_5_ (99.99%) were weighed according to respective stoichiometric ratios. After uniform mixing via ball milling for 4 h with ethyl alcohol and zirconium balls, these slurries were dried and calcined at 1200 °C for 3 h in an air atmosphere. Then, the powders were ball milled again with smaller zirconium balls (1 mm in diameter) to obtain smaller particle sizes. After that, these slurries were dried, mixed with solution of poly(vinyl alcohol) (PVA), and pressed into disks with a diameter of 13 mm. Finally, after removing the binder at 800 °C for 2 h, the disks were sintered at 1230–1370 °C for 4 h.

### Structural Characterization

XRD (Bruker, D8 ADVANCE for variable temperature tests and D2 PHASER for room temperature tests) with Cu Ka radiation (*λ* = 1.5406 Å) and Raman spectroscopy (Renishaw inVia reflex) were used to determine the crystal structure. The microstructure was recorded by a field emission scanning electron microscope (FE‐SEM, S‐4800, Hitachi, Tokyo, Japan). Spectroscopic ellipsometry (SE) measurements was used to measure the optical dielectric constant in the photon energy range of 1.24–4.96 eV (1000–250 nm) (V‐VASE by J. A. Woollam Co., Inc.), and a three‐layer structure model (air, rough layer, and ceramics) is used to fit the spectra. The response of domains were studied by a commercial AFM systen (Jupiter XR, Oxford, UK). The selected area electron diffraction and domain morphology observation were performed using a JEM‐2100F filed‐emission transmission electron microscope (FE‐TEM, JEOL, Japan).

### Properties Measurement

The *P*–*E* loops were measured by a ferroelectric measuring system (aixACCT TF Analyzer 2000E) with the sample size of 0.08 mm (thickness) × 0.785 mm^2^ (Au electrode area). The temperature‐ and frequency‐dependent dielectric properties and complex impedance were measured by a broad frequency/temperature dielectric spectrometer (Novocontrol GmbH, Concept 80). The charge–discharge test under overdamped (with a load resistance of 100 Ω) and underdamped conditions were measured by a commercial charge–discharge platform (CFD‐001, Gogo Instruments Technology, Shanghai, China). The samples for the charge–discharge test were 0.1 mm in thickness with an Au electrode of 1.5 mm in diameter.

## Conflict of Interest

The authors declare no conflict of interest.

## Supporting information

Supporting Information

## Data Availability

The data that support the findings of this study are available from the corresponding author upon reasonable request.

## References

[1] L. Zhao , Q. Liu , J. Gao , S. Zhang , J. F. Li , Adv. Mater. 2017, 29, 1701824. 10.1002/adma.201701824 28628242

[2] L.‐F. Zhu , L. Zhao , Y. Yan , H. Leng , X. Li , L.‐Q. Cheng , X. Xiong , S. Priya , J. Mater. Chem. A 2021, 9, 9655.

[3] Z. Liu , X. Chen , W. Peng , C. Xu , X. Dong , F. Cao , G. Wang , Appl. Phys. Lett. 2015, 106, 262901.

[4] L. Chen , S. Deng , H. Liu , J. Wu , H. Qi , J. Chen , Nat. Commun. 2022, 13, 3089. 35654831 10.1038/s41467-022-30821-7 PMC9163056

[5] H. Qi , A. Xie , A. Tian , R. Zuo , Adv. Energy Mater. 2019, 10, 1903338.

[6] Q. Wang , B. Xie , Q. Zheng , M. A. Marwat , Z. Liu , P. Mao , S. Jiang , H. Zhang , Chem. Eng. J. 2023, 452, 139422.

[7] P. Zhao , Z. Fang , X. Zhang , J. Chen , Y. Shen , X. Zhang , Q. An , C. Yang , X. Gao , S. Zhang , B. Tang , ACS Appl. Mater. Interfaces 2021, 13, 24833. 34014637 10.1021/acsami.1c04274

[8] H. Pan , J. Ma , J. Ma , Q. Zhang , X. Liu , B. Guan , L. Gu , X. Zhang , Y. J. Zhang , L. Li , Y. Shen , Y. H. Lin , C. W. Nan , Nat. Commun. 2018, 9, 1813. 29739938 10.1038/s41467-018-04189-6 PMC5940880

[9] H. Pan , F. Li , Y. Liu , Q. Zhang , M. Wang , S. Lan , Y. Zheng , J. Ma , L. Gu , Y. Shen , P. Yu , S. Zhang , L. Q. Chen , Y. H. Lin , C. W. Nan , Science 2019, 365, 578. 31395780 10.1126/science.aaw8109

[10] K. Wang , J. Ouyang , M. Wuttig , Y.‐Y. Zhao , H. Cheng , Y. Zhang , R. Su , J. Yan , X. Zhong , F. Zeng , Adv. Energy Mater. 2020, 10, 2001778.

[11] H. Pan , S. Lan , S. Xu , Q. Zhang , H. Yao , Y. Liu , F. Meng , E. J. Guo , L. Gu , D. Yi , X. Renshaw Wang , H. Huang , J. L. MacManus‐Driscoll , L. Q. Chen , K. J. Jin , C. W. Nan , Y. H. Lin , Science 2021, 374, 100. 34591628 10.1126/science.abi7687

[12] L. Chen , N. Wang , Z. Zhang , H. Yu , J. Wu , S. Deng , H. Liu , H. Qi , J. Chen , Adv. Mater. 2022, 34, 2205787. 10.1002/adma.202205787 36063143

[13] F. Yan , K. Huang , T. Jiang , X. Zhou , Y. Shi , G. Ge , B. Shen , J. Zhai , Energy Storage Mater. 2020, 30, 392.

[14] Q. Hu , Y. Tian , Q. Zhu , J. Bian , L. Jin , H. Du , D. O. Alikin , V. Y. Shur , Y. Feng , Z. Xu , X. Wei , Nano Energy 2020, 67, 104264.

[15] A. Xie , R. Zuo , Z. Qiao , Z. Fu , T. Hu , L. Fei , Adv. Energy Mater. 2021, 11, 2101378.

[16] D. Hu , Z. Pan , X. Zhang , H. Ye , Z. He , M. Wang , S. Xing , J. Zhai , Q. Fu , J. Liu , J. Mater. Chem. C 2020, 8, 591.

[17] M. Zhang , H. Yang , Y. Lin , Q. Yuan , H. Du , Energy Storage Mater. 2022, 45, 861.

[18] L. Cao , Y. Yuan , Z. Yang , E. Li , S. Zhang , Ceram. Int. 2020, 46, 6108.

[19] X.‐J. He , Z.‐S. Xie , X. Yuan , L. Li , D.‐F. Huang , C.‐W. Tao , R.‐X. Wang , J. Hao , G. Yuan , S.‐T. Zhang , J. Am. Ceram. Soc. 2020, 103, 6913.

[20] M. P. Trubelja , E. Ryba , D. K. Smith , J. Mater. Sci. 1996, 31, 1435.

[21] I. A. Santos , D. U. Spínola , D. Garcia , J. A. Eiras , J. Appl. Phys. 2002, 92, 3251.

[22] X. Zhang , H. Wang , X. Bu , P. Zheng , L. Li , F. Wen , W. Bai , J. Zhang , L. Zheng , J. Zhai , Y. Zhang , Inorg. Chem. 2021, 60, 6559. 33861589 10.1021/acs.inorgchem.1c00362

[23] H. Wang , X. Bu , X. Zhang , P. Zheng , L. Li , F. Wen , W. Bai , J. Zhang , L. Zheng , Y. Zhang , ACS Appl. Energy Mater. 2021, 4, 9066.

[24] X.‐J. He , L. Li , Z.‐S. Xie , Y.‐C. Zhang , J. Zhang , Z.‐B. Gu , H. Zhang , G. Yuan , S.‐T. Zhang , J. Eur. Ceram. Soc. 2021, 41, 2435.

[25] Z. J. Yang , X. Q. Liu , X. L. Zhu , X. M. Chen , Appl. Phys. Lett. 2020, 117, 122902.

[26] J. Gardner , F. D. Morrison , Appl. Phys. Lett. 2016, 109, 072901.

[27] S. Hou , S. Xu , L. Yang , X. Liu , L. Wei , X. Chao , D. Wu , P. Liang , Z. Yang , Ceram. Int. 2022, 48, 28382.

[28] Y. Zhao , J. Wang , L. Zhang , X. Shi , S. Liu , D. Zhang , Ceram. Int. 2016, 42, 16697.

[29] B. Yang , J. Zhang , X. Lou , Y. Gao , P. Shi , Y. Yang , M. Yang , J. Cui , L. Wei , S. Sun , ACS Appl. Mater. Interfaces 2022, 14, 34855. 35867986 10.1021/acsami.2c06889

[30] K. Banerjee , N. Singh , S. Asthana , J. Phys. Chem. Solids 2022, 163, 110579.

[31] L. Yang , X. Kong , Q. Li , Y. H. Lin , S. Zhang , C. W. Nan , ACS Appl. Mater. Interfaces 2022, 14, 32218. 35816115 10.1021/acsami.2c05205

[32] X. Zhu , M. Fu , M. C. Stennett , P. M. Vilarinho , I. Levin , C. A. Randall , J. Gardner , F. D. Morrison , I. M. Reaney , Chem. Mater. 2015, 27, 3250.

[33] Y. Wang , T. L. Sun , X. L. Zhu , L. Liu , J. Appl. Phys. 2021, 129, 244107.

[34] B. Yang , S. Hao , P. Yang , L. Wei , Z. Yang , Ceram. Int. 2018, 44, 8832.

[35] J. A. McNulty , D. Pesquera , J. Gardner , A. Rotaru , H. Y. Playford , M. G. Tucker , M. A. Carpenter , F. D. Morrison , Chem. Mater. 2020, 32, 8492.

[36] J. Gardner , F. D. Morrison , Dalton Trans. 2014, 43, 11687. 24950736 10.1039/c4dt00126e

[37] J. Gardner , F. Yu , C. Tang , W. Kockelmann , W. Zhou , F. D. Morrison , Chem. Mater. 2016, 28, 4616.

[38] X. Zhang , W. Ye , X. Bu , P. Zheng , L. Li , F. Wen , W. Bai , L. Zheng , Y. Zhang , Dalton Trans. 2021, 50, 124. 33305761 10.1039/d0dt03511d

[39] C. Zhu , W. Ye , P. Zheng , H. Zhang , F. Lu , Q. Fan , J. Zhang , L. Zheng , Y. Zhang , W. Bai , ACS Appl. Energy Mater. 2022, 5, 8492.

[40] Y. Zhou , S. Gao , J. Huang , M. Shen , S. Jiang , Y. He , Q. Zhang , J. Materiomics 2022, 9, 410.

[41] X. Li , Z. Tan , J. Xing , F. Wang , L. Xie , W. Zhang , N. Chen , H. Chen , J. Zhu , ACS Appl. Mater. Interfaces 2022, 14, 42245. 36074018 10.1021/acsami.2c11691

[42] L. Zheng , Z. Niu , P. Zheng , K. Zhang , C. Luo , J. Zhang , N. Wang , W. Bai , Y. Zhang , Mater. Today Energy 2022, 28, 101078.

[43] L. Chen , F. Li , B. Gao , C. Zhou , J. Wu , S. Deng , H. Liu , H. Qi , J. Chen , Chem. Eng. J. 2023, 452, 139222.

[44] Z. Wang , R. Kang , Z. Hong , X. Ke , X. Lou , L. Zhang , L. Zhang , J. Wang , ACS Appl. Mater. Interfaces 2022, 14, 44389. 36153962 10.1021/acsami.2c11871

[45] Z. Xu , Z. Liu , K. Dai , T. Lu , Z. Lv , Z. Hu , Y. Liu , G. Wang , J. Mater. Chem. A 2022, 10, 13907.

[46] C. Luo , C. Zhu , Y. Liang , P. Zheng , W. Bai , L. Li , F. Wen , J. Zhang , L. Zheng , Y. Zhang , ACS Appl. Electron. Mater. 2021, 4, 452.

[47] Y. Rao , H. Liu , H. Hao , Z. Yao , X. Zhou , M. Cao , Z. Yu , Ceram. Int. 2018, 44, 11022.

[48] X. Zhang , P. Zheng , L. Li , F. Wen , W. Bai , J.i Zhang , L. Zheng , Y. Zhang , Scr. Mater. 2022, 211, 114514.

[49] S. Xu , S. shen , R. Hao , Z. Peng , F. Zhang , D. Wu , P. Liang , X. Chao , L. Wei , Z. Yang , Chem. Eng. J. 2022, 433, 133812.

[50] S. Xu , R. Hao , Z. Yan , S. Hou , Z. Peng , D. Wu , P. Liang , X. Chao , L. Wei , Z. Yang , J. Eur. Ceram. Soc. 2022, 42, 2781.

[51] L. Cao , Y. Yuan , X. Meng , E. Li , B. Tang , ACS Appl. Mater. Interfaces 2022, 14, 9318. 35133128 10.1021/acsami.1c23673

[52] L. Cao , Y. Yuan , E. Li , S. Zhang , Chem. Eng. J. 2021, 421, 127846.

[53] L. Cao , Y. Yuan , X. Zhang , E. Li , S. Zhang , ACS Sustainable Chem. Eng. 2020, 8, 17527.

[54] M. Paściak , P. Ondrejkovic , J. Kulda , P. Vaněk , J. Drahokoupil , G. Steciuk , L. Palatinus , T. R. Welberry , H. E. Fischer , J. Hlinka , E. Buixaderas , Phys. Rev. B 2019, 99, 104102.

[55] R. Li , Y. Pu , Q. Zhang , W. Wang , J. Li , X. Du , M. Chen , X. Zhang , Z. Sun , J. Eur. Ceram. Soc. 2020, 40, 4509.

[56] T. Tunkasiri , G. Rujijanagul , J. Mater. Sci. Lett. 1996, 15, 1767.

[57] D. Li , D. Zhou , W. Liu , P.‐J. Wang , Y. Guo , X.‐G. Yao , H.‐X. Lin , Chem. Eng. J. 2021, 419, 129601.

[58] R. Kang , Z. Wang , W. Yang , X. Zhu , P. Shi , Y. Gao , P. Mao , J. Zhao , L. Zhang , X. Lou , J. Mater. Chem. A 2021, 9, 24387.

[59] K. Dai , A. Cui , Y. Ye , K. Jiang , J. Zhang , Y. Li , G. Wang , X. Dong , Z. Hu , J. Chu , Phys. Rev. B 2021, 104, 174104.

[60] H. T. Dang , T. T. Trinh , C. T. Q. Nguyen , T. V. Do , M. D. Nguyen , H. N. Vu , Mater. Chem. Phys. 2019, 234, 210.

[61] J. Shi , X. Chen , X. Li , J. Sun , C. Sun , F. Pang , H. Zhou , J. Mater. Chem. C 2020, 8, 3784.

[62] Y. Sun , S. A. Boggs , R. Ramprasad , Appl. Phys. Lett. 2012, 101, 132906.

[63] Y. Zhao , X. Liu , X. Zhang , H. Du , Materials 2022, 15, 4360. 35744422 10.3390/ma15124360 PMC9229208

[64] X. Dong , X. Li , X. Chen , Z. Tan , J. Wu , J. Zhu , H. Zhou , Nano Energy 2022, 101, 107577.

[65] X. Zhu , Y. Gao , P. Shi , R. Kang , F. Kang , W. Qiao , J. Zhao , Z. Wang , Y. Yuan , X. Lou , Nano Energy 2022, 98, 107276.

[66] X. Zhu , P. Shi , Y. Gao , R. Kang , J. Zhao , A. Xiao , W. Qiao , J. Zhao , Z. Wang , X. Lou , Chem. Eng. J. 2022, 437, 135462.

[67] B. Yang , L. Wei , X. Chao , Z. Wang , Z. Yang , J. Alloys Compd. 2015, 632, 368.

[68] F. Yan , H. Bai , G. Ge , J. Lin , C. Shi , K. Zhu , B. Shen , J. Zhai , S. Zhang , Small 2022, 18, 2106515. 10.1002/smll.202106515 35032092

[69] C. Xu , Z. Liu , X. Chen , S. Yan , F. Cao , X. Dong , G. Wang , J. Appl. Phys. 2016, 120, 074107.

