# Supplementary material for: Superior Energy Density Achieved in Unfilled Tungsten Bronze Ferroelectrics via Multiscale Regulation Strategy
Source: Adv Sci (Weinh). 2023 Apr 21;10(17):2300227. doi: 10.1002/advs.202300227 (PMC10265065; doi:10.1002/advs.202300227)
Supplement: Supplementary file 1 — Supporting Information [file ADVS-10-2300227-s001.pdf]

ADVANCED SCIENCE
Open Access

Supporting Information

for *Adv. Sci.*, DOI 10.1002/advs.202300227

Superior Energy Density Achieved in Unfilled Tungsten Bronze Ferroelectrics via Multiscale Regulation Strategy

Haonan Peng, Zhen Liu, Zhengqian Fu, Kai Dai, Zhongqian Lv, Shaobo Guo, Zhigao Hu, Fangfang Xu and Genshui Wang**

Superior Energy Density Achieved in Unfilled Tungsten Bronze Ferroelectrics via Multi-Scale Regulation Strategy

Supplementary Materials

Haonan Peng[1,2], Zhen Liu[1,], Zhengqian Fu[3], Kai Dai[4], Zhongqian Lv[1,2], Shaobo Guo[1], Zhigao Hu[4], Fangfang Xu[3], Genshui Wang[1,2,5 *]*

1, Key Laboratory of Inorganic Functional Materials and Devices, Shanghai Institute of Ceramics, Chinese Academy of Sciences, Shanghai 200050, People's Republic of China.

2, Center of Materials Science and Optoelectronics Engineering, University of Chinese Academy of Sciences, Beijing 100049, People's Republic of China.

3, State Key Laboratory of High Performance Ceramics and Superfine Microstructures, Shanghai Institute of Ceramics, Chinese Academy of Sciences, Shanghai 200050, China

4, Technical Center for Multifunctional Magneto-Optical Spectroscopy (Shanghai), Engineering Research Center of Nanophotonics & Advanced Instrument (Ministry of Education), Department of Materials, School of Physics and Electronic Science, East China Normal University, Shanghai 200241, China.

5, School of Chemistry and Materials Science, Hangzhou Institute for Advanced Study, University of Chinese Academy of Sciences, Hangzhou 310024, China.

The $Sr_{0.425}La_{0.1}\square_{0.05}Ba_{0.425}Nb_{2-x}Ta_xO_6$ ceramics with different Ta^{5+} doping (x=0.2, 0.4, 0.6, and 0.8) is studied to optimize the composition with best energy storage performance. All the components exhibit small size grains and a pure tungsten bronze structure revealed from the XRD patterns. With increasing Ta^{5+} content, the E_b and W_{rec} first rises and then declines. The sample of x=0.6 displays the largest E_b and a highest overall energy storage performance of W_{rec} = 5.895 J/cm^3 and η = 85.37%.

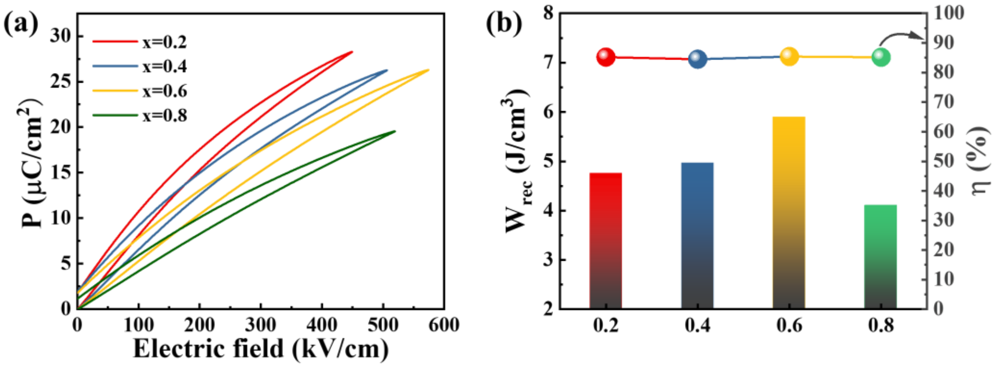

Figure S1. a) The *P*-*E* loops, b) W_{rec}, and η of $Sr_{0.425}La_{0.1}\square_{0.05}Ba_{0.425}Nb_{2-x}Ta_xO_6$ (x=0.2, 0.4, 0.6, and 0.8) under respective E_b.

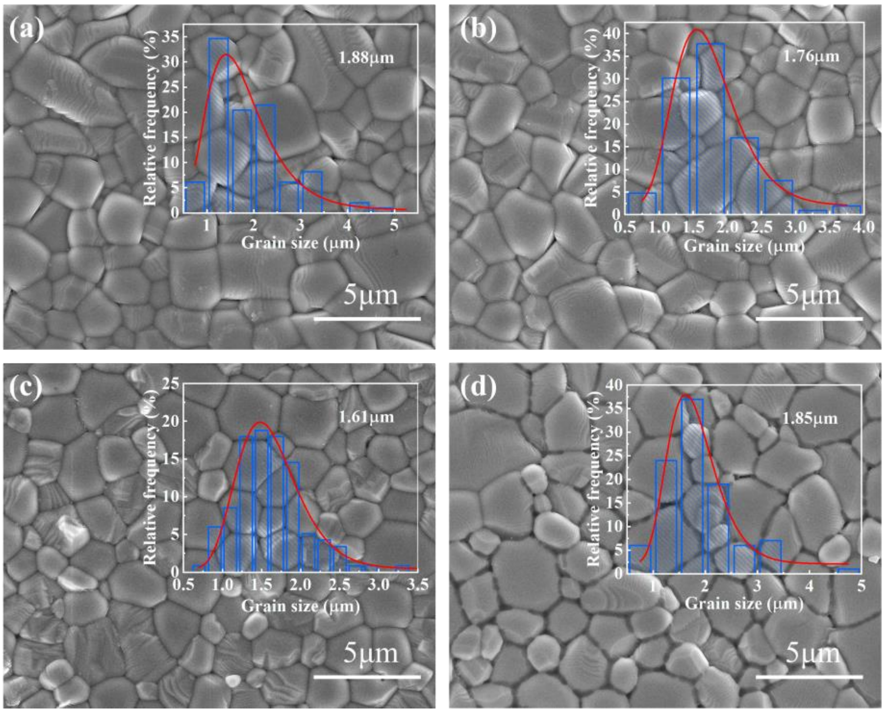

Figure S2. SEM images and grain size distributions of $Sr_{0.425}La_{0.1}\square_{0.05}Ba_{0.425}Nb_{2-x}Ta_xO_6$ ceramics, a)x=0.2, b)x=0.4, c)x=0.6, and d)x=0.8.

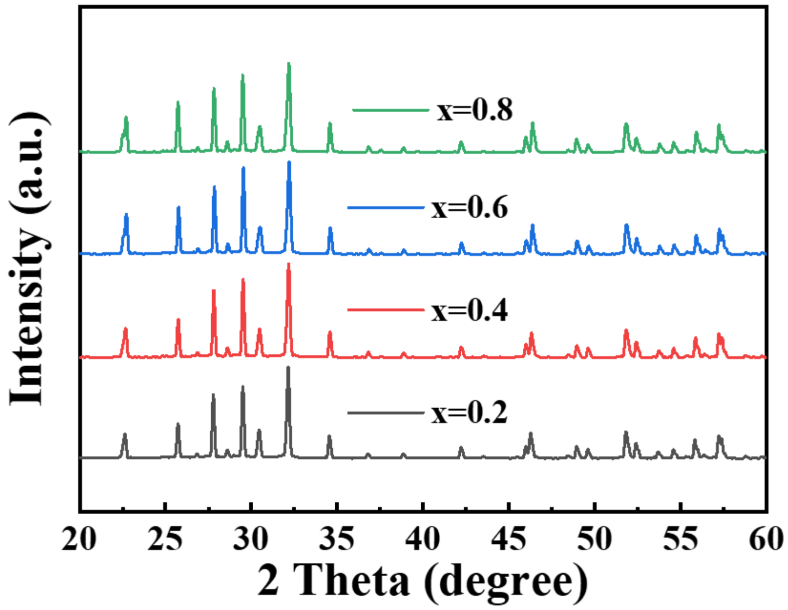

Figure S3. XRD patterns of the $Sr_{0.425}La_{0.1}\square_{0.05}Ba_{0.425}Nb_{2-x}Ta_xO_6$ ceramics (x=0.2, 0.4, 0.6, and 0.8).

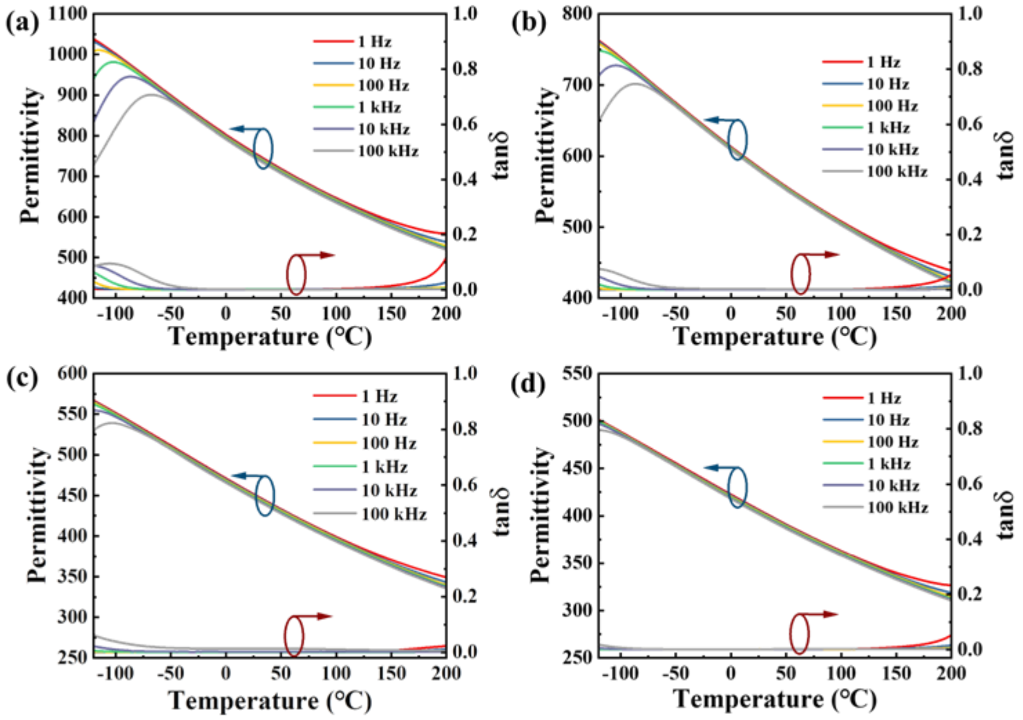

Figure S4. Temperature dependent permittivity and dielectric loss of $Sr_{0.425}La_{0.1}\square_{0.05}Ba_{0.425}Nb_{2-x}Ta_xO_6$ ceramics, a)x=0.2, b)x=0.4, c)x=0.6, and d)x=0.8.

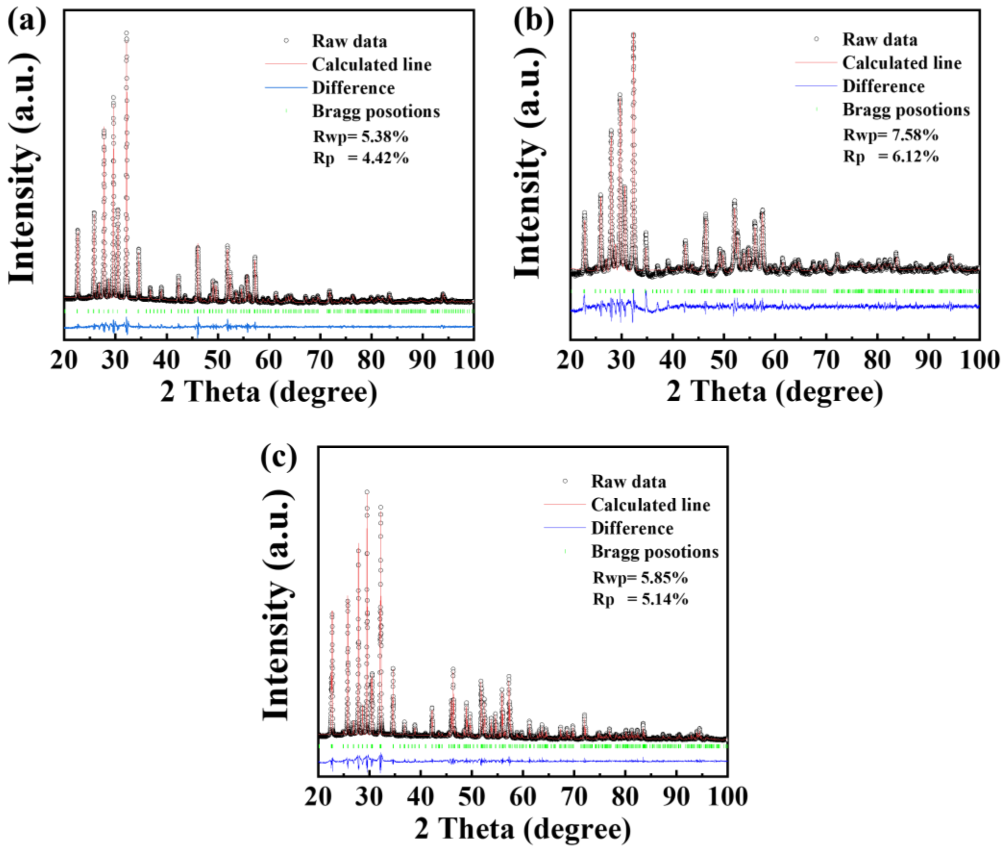

Figure S5. Rietveld refinement of the (a) SBN, (b) SLBN, and (C) SLBNT ceramics.

Table S1

Crystallographic data for the SBN, SLBN, and SLBNT ceramics calculated from the X-ray Rietveld refinements.

Formula	SBN	SLBN	SLBNT
**Space group**	*P4bm*	*P4bm*	*P4bm*
**a (Å)**	12.4722	12.4476	12.4743
**b (Å)**	12.4722	12.4476	12.4743
**c (Å)**	3.9460	3.9179	3.9157
**v ($Å^3$)**	613.8290	607.0440	609.3090
**c_{TTB}/a_{TTB}**	0.3164	0.3147	0.3139

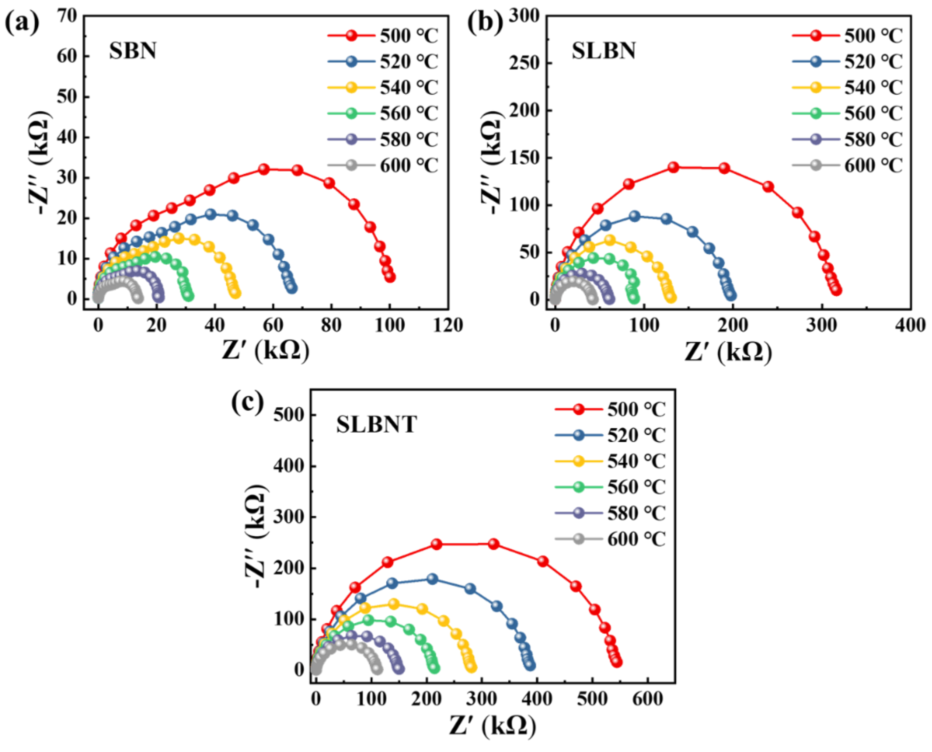

Figure S6. The impedance spectra of a) SBN, b) SLBN, and c) SLBNT ceramics.

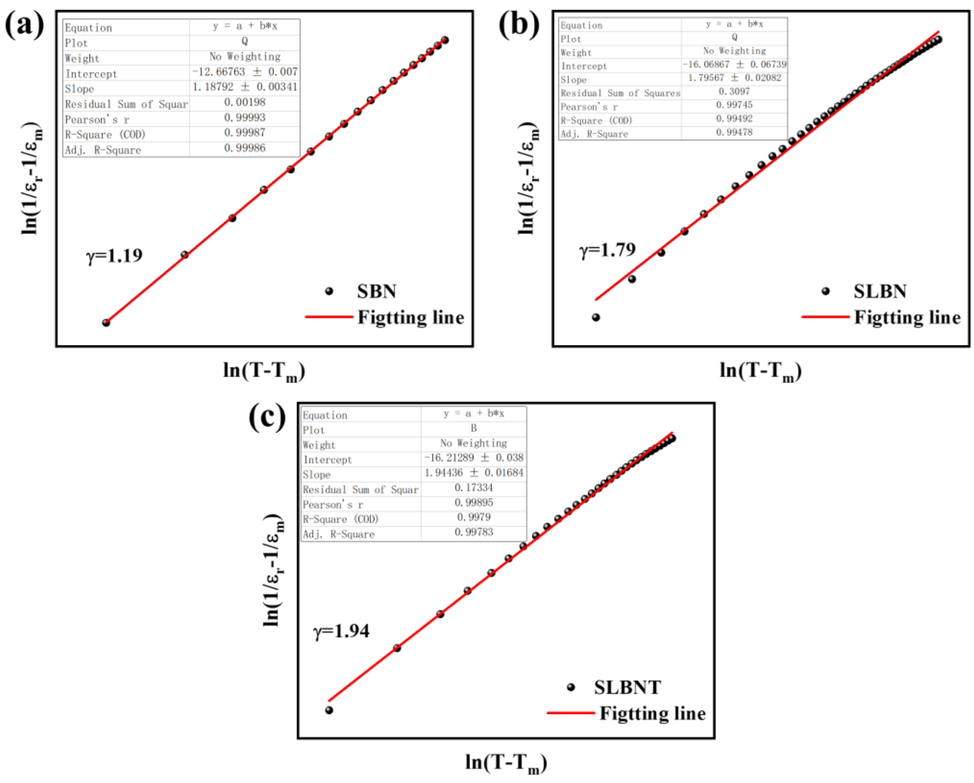

Figure S7. The diffuseness degree of SBN, SLBN, and SLBNT ceramics at 100 kHz.

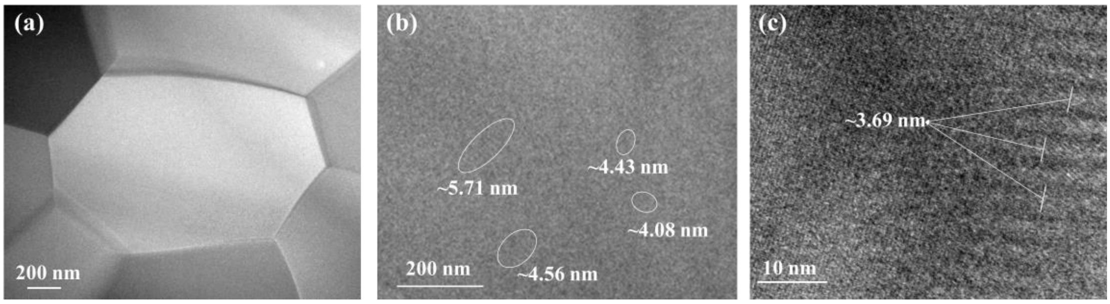

Figure S8. The TEM images at different magnifications of the domain morphology of SLBN ceramics.

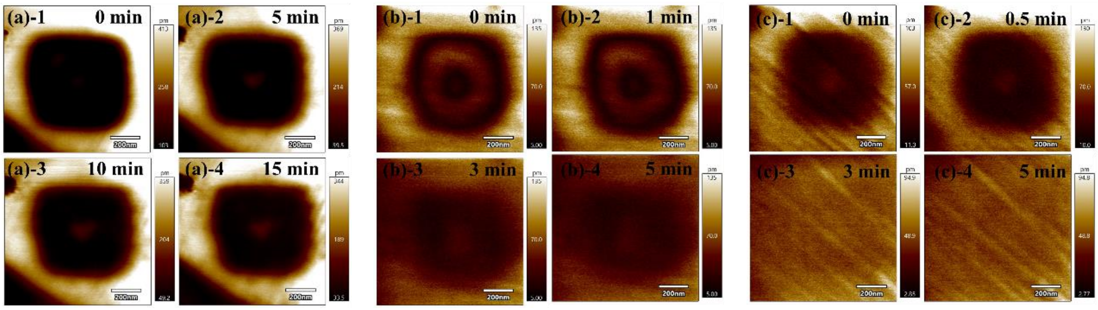

Figure S9. The out of plane amplitude image of (a)-1 to 4 SBN, (b)-1 to 4 SLBN, and (c)-1 to 4 SLBNT ceramics after a voltage of 15 V.

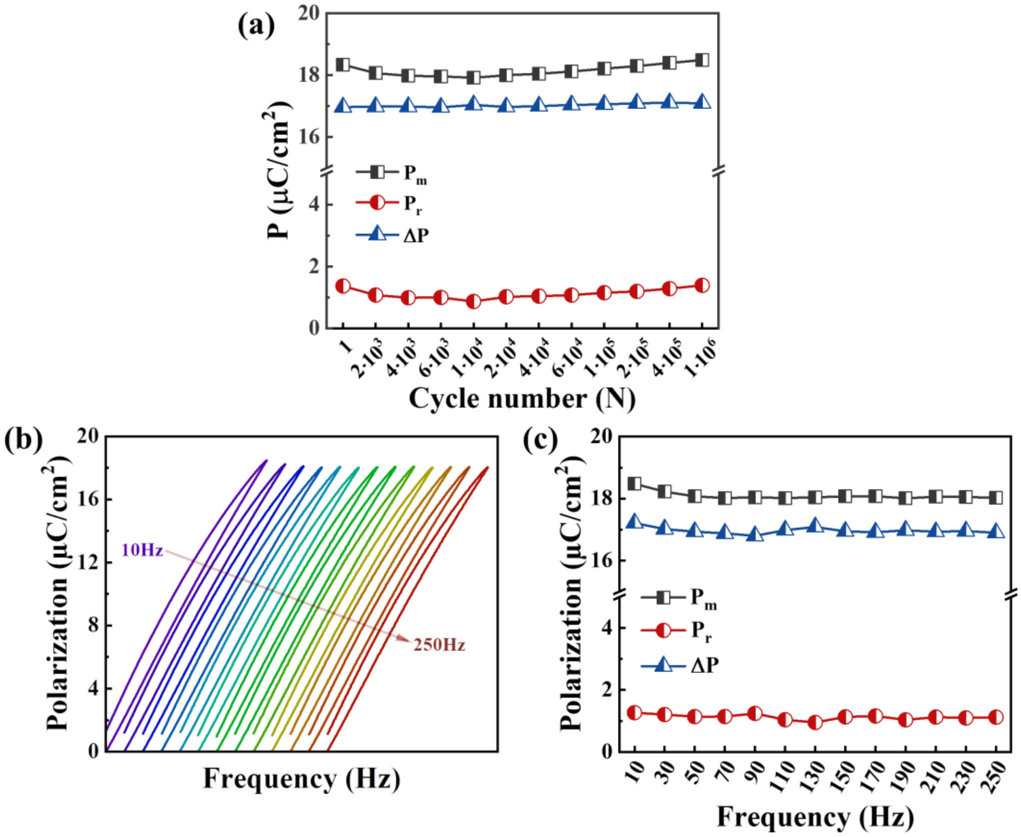

Figure S10. Polarization parameters (P_m, P_r, and ΔP) of SLBNT ceramics at different a) cycle numbers and c) frequencies. b) The *P-E* loops of SLBNT ceramics at different frequencies.
